# Aerosol in the oral health-care setting: a misty topic

**DOI:** 10.1007/s00784-023-05034-x

**Published:** 2023-05-10

**Authors:** Fridus Van der Weijden

**Affiliations:** Department of Periodontology, Academic Center for Dentistry Amsterdam (ACTA), University of Amsterdam and Vrije Universiteit Amsterdam, Gustav Mahlerlaan 3004, 1081 LA Amsterdam, The Netherlands

**Keywords:** Aerosol, Mouthrinse, Evacuator, Sars-CoV-2, Review

## Abstract

**Abstract:**

Studies have shown that mouth and respiratory tract microorganisms can be transported in aerosol and spatter. Due to aerosol-generating procedures, there are potentially various infection risks for patients and those working in health care, especially in oral health care. Dental aerosol can contaminate not only the mucous membranes of the oral health-care professional’s mouth, respiratory passages, and eyes but also exposed surfaces and materials in the environment. As such, preventing disease transmission within oral health-care offices is important issue. Since the start of the COVID-19 pandemic, an innumerable amount of (mis)information and advice on how to stay safe and prevent the spread of coronavirus has been published. What preventive measures can and have been taken to counteract this, and what have we learned during the pandemic? This review summarizes relevant literature that has addressed the presence and dispersal of aerosol and spatter as a concern in health care. It includes the sources of dental aerosol, their potential health threats, and strategies for controlling and mitigating their impact. It shows that further research is needed to better understand the potential health risks of dental aerosol and to develop effective strategies for mitigating them.

**Clinical relevance:**

Using personal protective equipment, high-volume evacuation systems and pre-procedural antimicrobial agents can help to reduce the potential for infection in oral health-care settings and protect the well-being of oral health-care workers and their patients.

## Introduction

Oral health care is generally classified in the very high-risk category of occupations involved with aerosol production, as the oral cavity is a habitat for microorganisms that can pose a risk for cross-contamination and infection [1]. Most dental procedures that use low- or high-speed handpieces, electrosurgery units, ultrasonic scalers, air polishers, prophy angles, hand instruments, air/water syringes, or lasers can create bio-aerosol [2]. Cooling instruments with water and air can create an aerosol with particles of < 1–5 µm, small enough to penetrate deep into the lungs [3]. As the oral environment is inherently wet with saliva that continuously replenishes the fluid in the mouth, the mouth is grossly contaminated with bacteria and viruses [4]. Studies demonstrate that microorganisms in the mouth and respiratory tract can be transported in the aerosol and spatter generated during dental procedures and can contaminate the skin and mucous membranes of the mouth, respiratory passages, and eyes of oral health-care personnel. Transmissions of airborne pathogens, therefore, pose a significant risk and have acquired special significance in the context of respiratory disease epidemics [5, 6]. The effect of aerosol on the aging population and immunocompromised patients in developed countries is also concerning due to their increased susceptibility to potentially infectious pathogens [7].

The potential routes for the spread of infection in an oral health-care clinic include direct contact with an infected patient’s body fluids, contact with environmental surfaces or instruments that the patient has contaminated, and contact with infectious particles from the patient that have become airborne [4]. In addition, an infectious microorganism may be transferred between the patient and members of the oral health-care team or from patient to patient from surfaces and materials exposed in the environment or instrument used during dental procedures. It is also possible that pathogens present in dental unit waterlines are spread through aerosol created by dental handpieces, presenting a risk for both the patient and members of the oral health-care team [1].

Over the past 3 years, aerosol has received considerable attention driven by fears of transmission of the severe acute respiratory syndrome coronavirus 2 (SARS-CoV-2 virus). Since its first identification in Wuhan, China, in November–December 2019, this virus has been implicated in the COVID-19 global pandemic. At the start of the pandemic, it was clear that the SARS-CoV-2 virus spreads via aerosol. The virus infection that resided deep in the lungs resulted in serious cases of pneumonia. Worldwide, it dawned on the oral health-care profession that they were possibly at increased risk of the SARS-CoV-2 virus spread. As a result, questions concerning the potential for the spread of infections from dental aerosol arose. The New York Times even suggested that dental hygienists face the greatest risk of SARS-CoV-2 virus infection, followed by dental assistants and dentists. At the beginning of the pandemic, oral health-care offices closed their doors, initially fueled by concerns that aerosol-generating dental procedures potentially increase the risk of transmitting respiratory pathogens through saliva [8].

While guidelines for cross-transmission prevention in oral health care are common in many countries, a tsunami of information about the SARS-CoV-2 virus and COVID-19 has appeared within the last 2.5 years. However, when reviewing the literature on COVID-19 infection prevention measures, it is striking that many recommendations need more scientific substantiation, and in almost all cases, an evaluation relating to possible risks is lacking [8].

Therefore, this paper aims to review relevant literature that has addressed the presence and dispersal of aerosol and spatter as a concern in health care due to their potential adverse health effects on patients and health-care workers. The review includes the sources of dental aerosol, their potential health threats, and strategies for controlling and mitigating their impact. The paper concludes with recommendations for the control of dental aerosol and spatter.

### Aerosol

An aerosol is a suspension of fine solid particles or liquid droplets in air or another gas. Particles have a residence time in air for more than a few seconds, are carried along with air currents, and can travel short distances before they settle on environmental surfaces or enter the respiratory tract [9]. Particles can consist of a single organism, a cluster of organisms, or organisms coagulated with body fluid or cells of skin or dust particles in the air. Aerosol-containing pathogens are considered infectious and responsible for airborne transmission of disease [10]. A concept that was first introduced by Wells in 1934 [11].

The definition of an aerosol can lead to confusion, as it varies widely according to the discipline describing it [6]. For instance, the standard medical use of the term “aerosol” to mean only particles ≤ 5 µm is not in line with modern aerosol physics [9]. But as the size of the droplets in an aerosol is an important factor in determining its potential for the transmission of infectious diseases, for this review, aerosol is classified based on where it deposits in the respiratory tract.

- *● Respirable aerosol* is defined as those particles (≤ 5 µm) small enough to penetrate and lodge into the smaller passages of the lungs and reach the respiratory bronchioles and alveoli. When containing pathogens, these small particles are highly capable of initiating a lower respiratory tract infection. These particles can also remain suspended in the air for long periods and can be dispersed over a wide area, increasing the potential for exposure to infectious agents.
- *● Thoracic aerosol* has larger particles (up to 10–15 µm) able to penetrate into the trachea and large intrathoracic airways.
- *● Inhalable aerosol* has the largest particles, up to about 100–200 µm, that can be aspirated into the nose.
- *●* Droplets > 200 μm that remain suspended in air or evaporate before reaching the ground may splash into mucus membranes. These particles differ from aerosol because they are visible to the naked eye and are considered too large to be inhaled and imposed deep within the lung. Spatter particles are ejected forcibly from the operating site and arc in a trajectory from the oral cavity until they contact a surface. Because of this trajectory, spatter particles do not remain suspended in the air for long periods, making them less likely to transmit disease via the airborne route.

When particles and aerosol droplets collide, they might disperse or coagulate, changing the particle size. In that case, the classifications described above no longer apply. These collisions randomly create a heterogeneous mixture of large and small particles with highly variable properties. Furthermore, environmental factors such as temperature, humidity, superimposition of new aerosol, and airflow further impact aerosol dynamics [12]. Which aerosol fraction-respirable, thoracic, or inhalable-is important depends on the agent and its target tissue. Only the respirable fraction is relevant if the target resides in the alveoli. However, in the case of a pathogen that uses a receptor that is present on the surface of cells throughout the length of the respiratory tract, all these aerosol fractions are likely to be important. In addition, because the mucous membrane is susceptible to SARS-CoV-2 infection, a spray hitting the eyes or the nostrils also poses a risk. The well-accepted risk of self-inoculation at these sites with fingers contaminated after touching surfaces should also be considered [9]. Both large and small aerosol particles may also contain blood elements with attached viral particles [4].

Another important consideration in cross-transmission is the viability of the microorganisms within the environment once they have left the host. When microorganisms leave their host and are aerosolized, they are potentially injured during the generation process [13]. Factors such as temperature, relative humidity, airflow, and oxygen sensitivity will impact whether microorganisms can survive outside the host and replicate. Early data indicate that microorganisms could remain viable in the airborne state for long enough to permit wide dissemination [14]

Cross-transmission occurs everywhere; however, transmission of pathogenic microorganisms does not necessarily result in infectious disease of the host. Volgenant and De Soet [5] propose a three-factor model that includes the risk of transmission, microorganism virulence, and exposure frequency to assess the relative infection risk. The factors involved can either increase or decrease the infection risk. The risk of transmission of pathogens in an oral health-care setting is still unknown but cannot be considered negligible as cross-transmission of microorganisms most likely occurs frequently. However, it appears that this does not often result in infections in the patient or oral health-care professional [5].

### Aerosol-generating procedures

Aerosol-generating procedures are defined as medical procedures that can result in the release of aerosol from the respiratory tract. The criteria for these procedures are a high risk of aerosol generation and an increased risk of respiratory transmission [15]. There is uncertainty around the levels of risk associated with various procedures because, in practice, there is no consensus on which are aerosol-generating. The current World Health Organization list of aerosol-generating procedures includes tracheal intubation, non-invasive tracheotomy, cardiopulmonary resuscitation, manual ventilation before intubation, bronchoscopy, sputum induction using nebulized hypertonic saline, autopsy, and dentistry procedures [16]. With the emergence of SARS-CoV-2, professional societies have unilaterally declared a plethora of additional procedures as aerosol-generating. Most of these designations were made on theoretical grounds rather than formal quantifications of aerosol generation or epidemiologic studies demonstrating an increased risk for infection [17].

In general, two categories of aerosol-generating medical procedures have been documented in the literature [6]:

- ● Those that induce the patient to express the contents of the lower respiratory tract by stimulating a cough reflex [sputum induction].
- ●Those that mechanically disrupt the contents of the respiratory tract.

A large body of evidence indicates that patients in the acute phase of respiratory infections can disseminate large numbers of airborne microorganisms. But aerosol is also generated during normal physiological activities such as breathing, talking, coughing, and sneezing [6]. Therefore, several source processes are recognized that can introduce aerosol and droplets with infectious material. These processes may occur simultaneously, each contributing incrementally to the total amount of suspended particles and resulting in mixtures from multiple sources and aerosol-generating procedures.

- *● Physiologically produced aerosol* is generated by patients and oral health-care personnel through talking, respiration, sneezing, and coughing in the clinical environment.
- *● Environmental sources of aerosol* from outside and inside the treatment room are present in oral health-care facilities as a result of heating, ventilation, air-conditioning, and airflow movement. With an inefficient ventilation system, the aerosol may be withheld or recycled. The treatment room environment may also contain previous aerosol particles that have settled on surfaces and become re-aerosolized.
- *● Treatment procedure aerosol* is composed of airborne fluids and particulates from the patient, along with coolant or irrigating solutions from instrumentation.

During oral health-care treatment procedures, the combination of high-speed instrumentation and a “wet environment” created by saliva and water coolant generates a large spray that disperses in many forms, as dictated by the physics of aerosol creation [6]. The production of airborne material during oral health-care procedures is evident to the oral health-care team and the patient. An aerosol cloud of particulate matter and fluid is often visible during dental procedures. This ubiquitous aerosolized cloud is a combination of materials originating from the treatment site and the dental unit waterlines [4]. In the dental literature, the terms “aerosol” and “spatter” were first used in the 1960s by Micik, Miller, and co-workers in their pioneering work on aerobiology [18, 19]. They examined characteristics of bio-aerosol generated from a patient’s mouth during dental procedures. Currently, according to the World Health Organization [16], the following aerosol-generating procedures are present in oral health care:

- ● All clinical procedures that use spray-generating equipment, such as three-way air/water spray, dental cleaning with an ultrasonic scaler and polishing, periodontal treatment with an ultrasonic scaler.
- ● Any kind of dental preparation with high- or low-speed handpieces.
- ● Direct and indirect restoration and polishing.
- ● Definitive cementation of crown or bridge.
- ● Mechanical endodontic treatment.
- ● Surgical tooth extraction and implant placement.

Exposure to dental aerosol has been associated with several health risks for both oral health-care professionals and their patients. For oral health-care professionals, exposure to a dental aerosol can lead to respiratory disorders such as asthma and chronic obstructive pulmonary disease (COPD). In addition, dental aerosol has been linked to an increased risk of infection from bloodborne pathogens [1]. Furthermore, exposure to certain materials in dental aerosol, such as mercury from dental amalgams, may lead to long-term adverse health effects [20]. The primary focus of this paper is on respiratory disorders, specifically SARS-CoV-2.

Multiple mechanisms are responsible for aerosolized and droplet particles exiting patients’ mouths. Fluid mixtures can splash against the soft and hard palate, tooth, tongue, or gingiva to create droplets. Fluids can also contact high rotational speed instruments, causing rapid directional changes in the fluid flow and droplet momentum, turbulent mixing of air and fluid, and disruption of fluid surface tension, generating large numbers of aerosol and droplet particles. These mechanisms typically occur whenever handpieces and other powered dental instruments are used in procedures. The distribution of contaminated aerosol and spatter is highly variable and may be influenced by many factors. These include the type of procedure and whether high-volume evacuation was used; the tools and equipment used; the pressure and flow rate of the water or air; the position of the tooth in the mouth, which affects the position of the operator relative to the subject; the position of the subject in the dental chair; the levels of microorganisms in the subject’s mouth; and operator handedness and position [7].

The microbial load of dental aerosol may also vary widely depending on its source [21]. Estimates put the ratio of saliva to water coolant in dental aerosol from all devices between 1:20 and 1:100 [6]. This could imply that 95–99% of aerosol is composed of water coolant. The implications are that coolant water contamination should not be overlooked and that oral or respiratory pathogen content is diluted from levels found in saliva or blood if the mixture is homogenized. In addition to dental unit water, the air delivered by rotary handpieces and air/water syringes is also a significant contributor to the formation of aerosol.

Currently, no direct evidence suggests that transmission of the SARS-CoV-2 virus is associated with any specific dental procedures. However, the SARS-CoV-2 virus and antibodies have been found in infected patients’ saliva [22]. In addition, the proximity between patient and oral health-care personnel during treatment where exposure directly from aerosol occurs [23] suggests a plausible risk of SARS-CoV-2 transmission associated with dental treatment.

### Measures in the oral health to prevent aerosol-generated infections

Effective infection control measures are essential to prevent the transmission of infectious diseases through dental aerosol. By implementing proper infection control measures and utilizing chemical agents, oral health-care workers can reduce this risk and ensure their own safety, and the safety of their patients and others in the oral health-care clinic. Available guidelines for infection prevention evolve as and when the epidemiology of the emerging infection becomes more specific, and controversies are resolved [10].

The National Center for Chronic Disease Prevention and Health Promotion (CDC) has consolidated recommendations for preventing and controlling infectious diseases and managing personnel health and safety concerns related to infection control in oral health-care settings in “Guidelines for Infection Control in Dental Health-Care Settings” (2003) [2]. In 1993, CDC recommendations regarding infection control for oral health care were primarily set on theoretical rationale and expert opinion to reduce the risk of transmission of bloodborne pathogens among oral health-care personnel and patients. The universal precautions were based on the concept that all patients may be asymptomatic carriers of bloodborne pathogens or unaware they are infected [21]. Later, in 1996, the CDC revised the recommendations and adopted the term “standard precautions.” Standard precautions embrace the standard of care provided to minimize the risk of disease transmission to the oral health-care professional and patients through pathogens in blood and other body fluids, including saliva and respiratory secretions, as potentially infectious material. The oral health-care team should not rely on a single precautionary strategy. In addition to standard precautions, other measures might be necessary to prevent the potential spread of certain diseases that are transmitted through airborne, droplet, or contact transmission.

The best way to prevent airborne transmission is to stop the pathogen from escaping the immediate treatment site. Controls closer to the source are potentially more effective as they stop aerosol and droplet clouds. One of the most effective measures is the use of high-volume evacuation systems, which can effectively remove aerosol particles generated from the immediate treatment area. Another important measure is the use of antimicrobial agents to reduce the concentration of microorganisms in the aerosol.

Personal protective equipment is the farthest from the source, positioned just before an oral health-care professional’s body is exposed to the hazard. The primary recommendation for personal protective measures includes wearing a surgical mask and eye protection with solid side shields or a face shield to protect mucous membranes of the eyes, nose, and mouth during procedures likely to generate splashing or spattering of blood or other body fluids. Other recommendations include wearing protective clothing (e.g., reusable or disposable gown, laboratory coat, or uniform) that covers personal clothing and skin likely to be soiled with blood, saliva, and other potentially infectious materials; wearing medical gloves and sterile surgeon’s gloves when performing oral surgical procedures; ensuring strict adherence to hand hygiene and cough etiquette; disinfection and sterilization of dental instruments; and environmental disinfection to mitigate the risk of droplet- and contact-based transmission.

Combining high-volume evacuation systems and personal protective equipment with antimicrobial agents will help achieve the best possible results [4]. It is also important to properly disinfect and sterilize dental equipment and surfaces. However, these recommendations are not sufficient to completely eliminate the risk of infection transmission through dental aerosol. Therefore, oral health-care providers must remain vigilant and follow infection control guidelines to help reduce the presence of microorganisms in dental aerosol and protect themselves and their patients.

### High-volume evacuation systems

While there are currently no practical or economical source controls that completely contain dental aerosol and droplets, from a practical point of view, controls close to the source, such as high-volume evacuation devices, are generally effective. They stop aerosol and droplet clouds from dispersing over larger clinical areas and settling on clinical surfaces with which oral health-care personnel or patients make direct contact.

These systems typically consist of a handpiece with a saliva ejector and a hose connected to a vacuum source. By creating a strong suction force, high-volume evacuation devices can effectively capture and remove aerosol before they have a chance to become airborne. However, it is important to note that high-volume evacuation devices will not efficiently attract or reduce spatter and large droplets from the operating field as they have higher mass and kinetic energy to resist the airflow [19]. Furthermore, the high-volume evacuation system should preferably not be released into the operatory. Nevertheless, the high-volume evacuator provides an efficient way to capture aerosol for two reasons:

- ● The large diameter tubes and larger internal diameters offer less resistance to airflow, so the vacuum flow rate can be higher.
- ● The tip of the high-volume evacuator can be placed close to the location where aerosol is generated.

Research has demonstrated that the number of colony-forming units (CFU) produced during dental procedures is significantly reduced when an assistant uses a high-volume evacuation device [18, 25]. However, a problem arises when the operator works without an assistant, which is often the case during the delivery of periodontal treatment by a dental hygienist [26]. An option for oral health-care workers working without an assistant is using the operating instrument in one hand and the high-volume evacuation device in the other hand.

A study by Timmerman et al. [27] determined the atmospheric microbial contamination during initial periodontal treatment using a piezoelectric ultrasonic scaler in combination with a high-volume evacuation device. Based on the index of microbial air contamination [28], Timmerman and colleagues’ results indicate that the operatory atmosphere is considered to be in good condition during 40 min of continuous use of the ultrasonic scaler [27]. A more recent study evaluated the capture efficiency of aerosol and spatters from ultrasonic scalers with a high-volume evacuation device [29]. The ultrasonic scaling procedure generated a wide size range of aerosol particles (up to a few hundred mm) and occasional large spatters, which were emitted at low velocity (mostly < 3 m/s). Using a high-volume evacuator resulted in an overall reduction of 88%.

Furthermore, the presence of SARS-CoV-2 in aerosol from patients with COVID-19 was investigated as associated with ultrasonic scaling and tooth preparation [30]. A high-volume suction capacity (air volume) of 150 mm Hg or 325 L/min appeared sufficient for the elimination of viral contamination. The type of cannula or tip used can affect the flow rate and effectiveness. The small opening of a saliva ejector does not remove a large enough volume of air to be classified as high-volume suction [4]. Earlier work has shown that combining a high-volume evacuation device and a pre-procedural rinse reduces dental bio-aerosol more efficiently than each method individually [31].

### Pre-procedural rinsing

The intended effect of pre-procedural rinsing is to reduce the microbiological and viral pressure in the aerosol. In the past, studies have evaluated the ability of pre-procedural rinses to reduce the number of CFUs produced by various dental instruments [4]. A recent Cochrane review [32] summarized the literature on the effect of pre-procedural mouth rinsing in preventing aerosol transmission of infectious diseases in oral health-care providers. The authors conclude that overall, the results suggest that using a pre-procedural mouthwash may reduce the level of bacterial contamination in aerosol compared to no pre-rinse or water rinsing. Earlier studies identified a potential anti-bacterial effect of povidone-iodine, chlorhexidine, and cetylpyridinium chloride [33, 33, 34].

As the SARS-CoV-2 virus has been detected in infected patients in saliva [22], pre-procedural rinsing is a preventive action to limit the viral load in the saliva in asymptomatic or pre-symptomatic patients [35]. Possibly, the earliest paper to suggest that mouthwashes are a potential way to reduce transmission of SARS-CoV-2 was published in early May 2020 [36]. A recent study [37] indicates that in the case of pre-procedural rinsing, the air in the treatment room does not contain SARS-CoV-2 virus particles, as was evaluated in the HEPA filter of an air purifier. While not all researchers agree [38], recent systematic reviews describe the effect of various mouthwash agents on the SARS-CoV-2 virus. Mouthwash ingredients that appear to be potentially most effective are the povidone-iodine solution, hydrogen peroxide, chlorhexidine, and cetylpyridinium chloride [39, 40].

#### Povidone-iodine

The effect of a povidone-iodine solution was evaluated separately in a systematic review in 2022 [41]. Based on four randomized controlled trials, the authors conclude that an iodine solution is effective against SARS-CoV-2 in the saliva. Therefore, the authors recommend rinsing with a povidone-iodine solution before treatment in a health-care facility to reduce the risk of cross-contamination. However, some reservation is needed as povidone-iodine may cause allergic contact or irritant dermatitis. Although this is a rare phenomenon, there are several case reports of generalized urticaria and even anaphylactic shock [42].

#### Hydrogen peroxide

A recent systematic review found no indication that pre-procedural rinsing with 1% hydrogen peroxide will reduce the viral load of SARS-CoV-2 in saliva [43]. According to a systematic, living review [44], the effect of hydrogen peroxide is not significant. However, a study in which SARS-CoV-2 positive patients rinsed for 1 min did measure a reduction with hydrogen peroxide [45]. Hydrogen peroxide is rapidly degraded in the mouth, which means it quickly loses its effectiveness [46]. The effect mainly occurs during rinsing and disappears shortly after expectorating [47]. An “in vivo” pilot study demonstrated that hydrogen peroxide suppresses the viral pressure of the SARS-CoV-2 virus in saliva for up to 30 min after use [48].

#### Chlorhexidine

Regarding reducing bacteria in an aerosol, a network analysis published in 2020 shows that pre-rinsing with chlorhexidine ranks as the most effective treatment for reduced postprocedural bacterial load [49]. A systematic review of chlorhexidine substantiates the antiviral properties of this mouthwash [50]. Another systematic review and meta-analysis reviewing types of mouthwashes and nasal sprays [51] concludes that, after a povidone-iodine solution, chlorhexidine is the most effective in vivo to reduce the viral load of SARS-CoV-2 in saliva. Research shows that the substantivity of chlorhexidine is at least 4 h [52] and that the efficacy lasts longer than with, for example, hydrogen peroxide.

#### Cetylpyridinium chloride [CPC]

There is no separate systematic review of CPC in which the antiviral properties of this mouthwash are substantiated. Ex vivo research indicates that a CPC mouthwash is effective in inactivating SARS-CoV-2 alpha to delta variants in saliva [53]. Even low concentrations of CPC have been shown to suppress the infectivity of SARS-CoV-2 strains (Wuhan, Alpha, Beta, and Gamma) isolated from humans [54]. A systematic review in 2022 evaluated several types of mouthwash [55] and concluded that, after a povidone-iodine solution, CPC is the most effective in vitro and in vivo to reduce the viral load of SARS-CoV-2 in saliva. CPC has a substantivity of 2–5 h [56]. In vivo, research demonstrates that the viral pressure of the SARS-CoV-2 virus in the saliva is suppressed for up to 3–6 h after rinsing with CPC [57, 58].

#### Miscellaneous

Although less extensively studied, “in vitro” research shows that the SARS-CoV-2 virus in saliva is reduced by Listerine [59], octenidine mouthwash [60], D-limonene and CPC mouthwash [62 and 63, 64], and also stannous fluoride toothpaste [64], and also shown from “in vivo” studies with CPC and zinc mouthwash [48].

### Final considerations

Mouthwashes are a simple and effective way to reduce the viral load in saliva. However, whether this leads to a decrease of SARS-CoV-2 in the aerosol and whether it is related to a reduction in the risk of infection is debatable. Therefore, even though the effort invested in studying dental aerosol is reasonable, these studies do not include causal relationships that preclude robust decision-making. One of the gaps in knowledge is whether saliva is the main source of the aerosolized microbiota in oral care. The source of aerosols in the oral health-care setting is complex, with contributions from multiple sources. The exact contribution of each source may vary depending on factors such as the type of dental procedure, the equipment used, and the maintenance of the dental unit’s water lines. There is plausible evidence to suggest that water might contribute to a large portion of the microbial load in dental aerosol. Meethil et al. [65] performed an instrumentation study using preoperative mouthrinses and intraoral high-volume evacuation. They discovered that 78% of the microbiota in condensate could be traced to the dental irrigant, while saliva contributed to a median of 0% of aerosol microbiota. Although they identified low-copy numbers of the SARS-CoV-2 virus in the saliva of asymptomatic patients, none was found in aerosol generated from these patients. Within the limitations of their research, the bacterial and viral data indicate that when adequate infection control measures are used, dental treatment is not a factor in increasing the risk for transmission of SARS-CoV-2 in asymptomatic patients. This is further supported by the fact that water as a coolant has a typical flow rate of 10 to 40 mL per minute, whereas the flow rate of saliva during the same period is 0.4–0.5 mL. Thus, as previously stated, the estimated dilution ratio varies between 1:20 to 1:100 [6]. Onoyama et al. [66] found that the majority of droplet sizes generated from dental devices were larger than 50 µm. Aerosol-generating dental procedures emitted few aerosol particles that were smaller than 5 µm, a size that is a concern for pulmonary infections due to airborne transmission.

In future research, it is therefore important to conduct detailed studies that distinguish between microorganisms originating from saliva or other parts of the oral cavity and those present in coolant water or the environment. There is currently a lack of standardized protocols for evaluating microorganisms in dental aerosol which may explain inconsistencies in results across studies as isolation and determination is challenging [67]. For example, small aerosol particles can remain suspended in the air for extended periods of time, making them difficult to capture and isolate. Also, the concentration of microorganisms in dental aerosol is generally low, which can make detection and quantification challenging. Additionally, viruses can be negatively affected by heat and drying, potentially leading to false negative results during sample collection and processing [68]. There is also a risk of contamination during the collection and handling of aerosol samples. Yet, the need to differentiate between sources of microorganisms is a crucial issue in dental infection prevention.

The assumption that the oral health-care profession has an increased risk of aerosol-related infections has been questioned by the results of a recent retrospective analysis from the Netherlands. Based on the data from almost two million tests for those experiencing symptoms compatible with COVID-19, dentists and dental hygienists do not test SARS-CoV-2 positive any more often than other health-care workers or those with a non-close-contact occupation [8]. Furthermore, cluster outbreaks in oral health-care clinics have not yet been reported, which may indicate that aerosol-generating dental procedures do not pose a significant threat in contributing to the spread of the SARS-CoV-2 virus [66]. In the rush to be cautious, the basic fact that for decades oral health-care professionals have routinely generated aerosol in patients infected by pathogens seems to have been lost. Using high-volume evacuation and routine personal protective equipment has protected oral health-care providers, staff, and patients from cluster infections. If this were not the case, there would be ample evidence to the contrary. Standard infection control practices, as described above, appear sufficiently capable of protecting personnel and patients from exposure to potential pathogens. For the future, there will be a need to distinguish between meaningful or inactive infection preventive measures to be ready for another pandemic. Then, hopefully, the oral health care will not have to close its doors again.

## Conclusion

Dental aerosol is a potential source of transmission for infectious diseases. Further research is needed to better understand the characteristics of dental aerosol and the measures that can be taken to minimize their potential for the transmission of infection. Using personal protective equipment, high-volume evacuation systems and pre-procedural antimicrobial agents can help to reduce the potential for infection in oral health-care settings and protect the well-being of oral health-care workers and their patients. Further research is needed to better understand the potential health risks of dental aerosol and to develop effective strategies for mitigating them.
